# Internalised Weight Stigma Mediates Relationships Between Perceived Weight Stigma and Psychosocial Correlates in Individuals Seeking Bariatric Surgery: a Cross-sectional Study

**DOI:** 10.1007/s11695-022-06245-z

**Published:** 2022-09-12

**Authors:** Hugh Bidstrup, Leah Brennan, Annemarie Hindle, Leah Kaufmann, Xochitl de la Piedad Garcia

**Affiliations:** 1 School of Behavioural and Health Sciences, Australian Catholic University, 115 Victoria Parade, Fitzroy, VIC 3065 Australia; 2 School of Psychology and Public Health, La Trobe University, Albury-Wodonga, 3690 Australia; 3 Centre for Eating, Weight, and Body Image, Suite 215, 100 Victoria Parade, East Melbourne, VIC 3002 Australia

**Keywords:** Internalised weight stigma, Mediation, Bariatric surgery, Disordered eating, Quality of life, Depression, Anxiety

## Abstract

**Purpose:**

Research suggests that internalised weight stigma may explain the relationship between perceived weight stigma and adverse psychological correlates (e.g. depression, disordered eating, body image disturbances). However, few studies have assessed this mechanism in individuals seeking bariatric surgery, even though depression and disordered eating are more common in this group than the general population.

**Materials and Methods:**

We used data from a cross-sectional study with individuals seeking bariatric surgery (*n* = 217; 73.6% female) from Melbourne, Australia. Participants (*M*_age_ = 44.1 years, *SD* = 11.9; *M*_BMI_ = 43.1, *SD* = 7.9) completed a battery of self-report measures on weight stigma and biopsychosocial variables, prior to their procedures. Bias-corrected bootstrapped mediations were used to test the mediating role of internalised weight stigma. Significance thresholds were statistically corrected to reduce the risk of Type I error due to the large number of mediation tests conducted.

**Results:**

Controlling for BMI, internalised weight stigma mediated the relationship between perceived weight stigma and psychological quality of life, symptoms of depression and anxiety, stress, adverse coping behaviours, self-esteem, exercise avoidance, some disordered eating measures and body image subscales, but *not* physical quality of life or pain.

**Conclusion:**

Although the findings are cross-sectional, they are mostly consistent with previous research in other cohorts and provide partial support for theoretical models of weight stigma. Interventions addressing internalised weight stigma may be a useful tool for clinicians to reduce the negative correlates associated with weight stigma.

**Graphical abstract:**

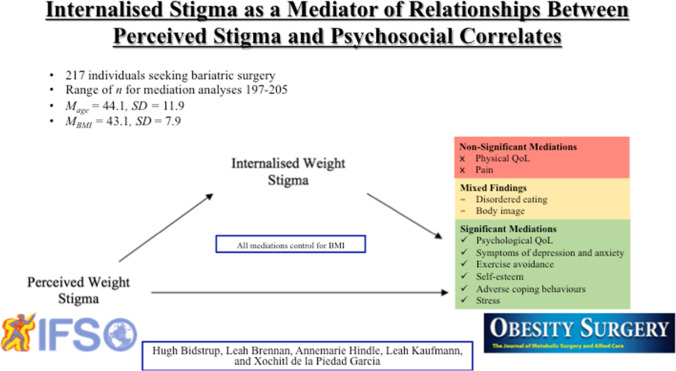

**Supplementary Information:**

The online version contains supplementary material available at 10.1007/s11695-022-06245-z.

## Introduction

Weight stigma is the pervasive social devaluation enacted towards individuals because of their weight [1]. Individuals living with higher weight report experiencing weight stigma between one and six times per week [2–4]. From the perspective of these individuals, weight stigma can manifest as stigma (a) experiences (e.g. being called names), (b) perceptions (e.g. feeling like others are staring at you), (c) internalisation (i.e. where individuals apply negative stereotypes about weight to themselves resulting in self-devaluation—e.g. believing you are not worthy of love or a job), and (d) anticipation (e.g. expecting poor treatment from others) [5–7]. A recent meta-analysis found a significant moderate association between public (i.e. perceived and experienced; *k* = 241) weight stigma and internalised weight stigma (*k* = 222) and adverse psychological health [8]. Research has found these associations across community, clinical, and student cohorts, and also in bariatric surgery patients and individuals seeking bariatric surgery [9, 10].

Meta-analytic evidence indicates that depression and binge eating disorder are more common in bariatric surgery patients and individuals seeking bariatric surgery compared to the general population [11]. Moreover, cross-sectional studies with individuals seeking bariatric surgery have found that experienced weight stigma is associated with emotional eating, body shame, internalised shame, and lower self-compassion [12], and that internalised weight stigma is associated with depression, anxiety, and poor quality of life and self-esteem [13]. However, the precise mechanism(s) through which weight stigma is associated with adverse correlates in this population has been understudied.

Some scholars have suggested the extent to which individuals *internalise* weight stigma mediates the relationships between experienced/perceived weight stigma and psychosocial correlates [14, 15]. A recent systematic review [16] indicated only one paper had assessed this mechanism in individuals seeking bariatric surgery, with mixed results [17]. Internalised weight stigma did not significantly mediate the relationship between experienced weight stigma and depression or anxiety when measured as the *sole* mediator. However, it was a significant mediator in models that also included other mediators such as body shame, internalised shame, and self-compassion. Thus, given the observed differences between bariatric surgery populations and the general population [11], there is a need to further investigate the mechanisms underpinning the relationship between weight stigma and psychosocial correlates in individuals seeking bariatric surgery, with a specific focus on internalised weight stigma as a mediator. Identifying the mechanisms through which weight stigma is associated with adverse correlates may provide support to existing theoretical models from which targeted clinical interventions can be developed and implemented as part of routine pre and post bariatric surgery care.

The aims of the current study were to estimate (a) the relationship between both perceived and internalised weight stigma, and psychosocial and physical correlates, and (b) the indirect effect of perceived weight stigma on the relationship between psychosocial and physical health correlates, via internalised weight stigma [14]. Based on previous research [8], we expected to find (a) a positive correlation between perceived and internalised weight stigma, (b) correlations between perceived and internalised weight stigma and negative psychosocial correlates (e.g. higher levels of depression, lower levels of quality of life), and (c) that internalised weight stigma would mediate the relationship between perceived weight stigma and psychosocial correlates [16]. Lastly, we made no prediction regarding physical health correlates, as research on physical health correlates in this domain is, at present, exploratory.

## Method

### Participants

Participants (*n* = 217; 73.6% female) were recruited prior to their procedures between 2014 and 2015 from a private bariatric surgery clinic located in Melbourne, Australia (*M*_age_ = 44.1 years, *SD* = 11.9; *M*_BMI (kg/m2)_ = 43.1, *SD* = 7.9). They were informed that participating would not affect their status as individuals seeking bariatric surgery. This was a cross-sectional study—all 217 participants in the current study were a subset of a larger study that completed weight stigma questionnaires, which were introduced after data collection began. Findings from this larger study have been published elsewhere [18]. As part of their consent, participants agreed to allow their non-identifiable, aggregated data to be used in future projects. Unfortunately, we do not have access to intake data that shows the number of individuals who were asked to participate but chose not to.

### Measures

#### Weight Stigma, Quality of Life, Disordered Eating, Body Image, Symptoms of Depression and Anxiety, and Other Biopsychosocial Measures

Table 1 shows all the measures used in the current study (excluding demographics). These include a perceived weight stigma scale, an internalised weight stigma scale, and a total of 16 psychosocial correlate scales. Some of the psychosocial correlate scales produce scores for subscales (total number of psychosocial correlate variables in the study is 51). For more detailed information on the scales, including descriptions, sample items, and reliability estimates, see the Measures section and Table S1 in the Supplementary Material.

**Table 1:** List of measures used in the current study

Scales used	Included global score/subscales, scale interpretations, and range of possible scores
Perception of Teasing Scale (POTS) [19]	General Weight Teasing Subscale. Items were modified to ask of current adulthood perceptions of teasing, not those of childhood as in the original scale. HS = frequent perceptions of teasing, 0–5
Weight Bias Internalisation Scale Modified (WBIS-11) [20]	Global score, HS = high internalised weight stigma, 1–7
Eating Disorder Examination Questionnaire (EDE-Q) [21, 22]	Global score and subscales on restraint, eating concern, weight concern, and shape concern. HS = high levels of disordered eating, 0–6
Dutch Eating Behaviour Questionnaire (DEBQ) [23]	Three subscales measuring restrained, emotional, and external eating. HS = high levels of disordered eating, 1–5
Three-Factor Eating Questionnaire (TFEQ) [24]	Three subscales measuring restrained eating, disinhibition, and perceived hunger. HS = high levels of disordered eating, 0–21/16/15, respectively
Clinical Impairment Assessment (CIA) [25]	Global score, HS = high levels of psychosocial impairment from disordered eating, 0–48
The Questionnaire on Eating and Weight Patterns (QEWP) [26]	Global score, HS = high levels of disordered eating, 13–58
Weight Efficacy Lifestyle Questionnaire (WEL) [27]	Five situational subscales: negative emotions, availability, social pressure, physical discomfort, and positive activities. HS = high self-efficacy in dietary restraint, 0–9
Exercise-Avoidance Motivation Scale (EAMS) [28]	Global score, HS = high levels of exercise avoidance, 1–7
Assessment of Quality of Life (AQoL) [29]	Two superdimensions of physical health (pain, senses, independent living) and mental health (happiness, mental health, coping, relationships, self-worth). HS = *good/better* QoL, 0–1
Impact of Weight on Quality of Life-Lite (IWQOL-Lite) [30]	Five subscales: physical function, self-esteem, sexual life, public distress, and work. HS = *poor* QoL, where weight negatively impacts QoL, 4–55, subscale dependent; 31–155 total score
The Generalized Anxiety Disorder Scale (GAD-7) [31]	Global score, HS = high levels of anxiety, 0–21
Depression Anxiety Stress Scales (DASS-21) [32]	Three subscales: depression, anxiety, and stress. HS = high levels of distress, 0–42
Patient Health Questionnaire (PHQ) [33]	HS = high levels of depressive symptoms, 0–27
Tolerance of Mood States Scale (TOMS) – Scale 2 [34]	From the recommendations in the scale development paper, we only assessed data from Scale 2 (explained in the Supplementary Material): maladaptive responses (general and eating) to intense moods. HS = more likely to respond to negative moods in a maladaptive way
Rosenberg Self-Esteem Scale (RSE) [35]	Global score, HS = high self-esteem, 0–30
The Multidimensional Body-Self Relations Questionnaire (MBSRQ) [36]	Ten subscales: appearance evaluation, appearance orientation, fitness evaluation, fitness orientation, health evaluation, health orientation, illness orientation, body areas satisfaction, overweight preoccupation, self-classified overweight. HS = see Table 5, 1–5
Brief Pain Inventory (BPI) [37]	Two subscales: the severity of pain and how pain interferes with daily life. HS = high levels of pain, 0–10

*Note. HS* high score on this scale indicates. A different research team selected all of these measures. We are simply reporting on data that has been already collected; thus, we have no rationale for why specific measures were used instead of others. For the POTS, we also computed the extent to which teasing upset the individual, where HS = teasing negatively affected the individual. Findings from this subscale of the POTS can be found in the Supplementary Material (Tables S4–S6)

#### Demographics

Participants were asked a series of demographic questions. These included questions asking participants’ age, sex, height and weight (for BMI), and birth country (see Table 2).

**Table 2:** Demographics of participants (*n* = 214) in the current study

	*M* or *n*	*SD* or %
Age (in years)	44.1	11.9
BMI	43.1	7.87
Gender (female)	153	73.6% female
Occupation		
Professional	62	29.0%
Manager	41	19.2%
Other (e.g. admin)	92	42.9%
Unemployed	11	5.1%
Not reported	8	3.7%
Birth country (Australia)	171	79.9% born in Australia
Education	116	55% tertiary educated

*Note.* Missing data ranged from 1 case (country and ethnicity) to 6 cases (gender). No measure had > 5% missing data. Ethnicity = Australian or Other, Birth County = Australia or Other, Education = tertiary educated (post school diploma, degree or above, or trade certificate) vs. not (completed secondary school until year 9, 10, 11, or 12)

### Procedure

Monash University’s Human Research Ethics Committee approved this research (CF11/0309 – 2011000106). Individuals seeking bariatric surgery were invited to participate by completing a pre-operative intake questionnaire. Participants completed the questionnaire in their own time and were provided with a postage paid envelope to return to the clinic when completed.

### Data Analysis Plan

Data cleaning and screening information can be found in the Supplementary Material. Of the 214 participants, 205 had complete data on measures of perceived and internalised weight stigma (see Table 1). The number of valid cases for our mediation analyses ranged between 198 and 205, depending on the psychosocial variable measured. First, we obtained descriptive statistics for all variables and conducted reliability analyses on all measures in SPSS [38]. Then, we obtained estimates of the bivariate relationships between perceived and internalised weight stigma, and psychosocial and physical correlates. Next, assumptions were checked. Although scores on the POTS were positively skewed, parametric tests are robust to violations of normality in large sample sizes [39, 40]. Leverage values did exceed the critical regions in most variables by 0.005–0.010; however, other similar metrics (e.g. Mahalanobis distance, Cook’s, DFBETAS, DFFITS) did not identify any influential cases. The data were then transferred to jamovi [41], where 51 separate bias-corrected bootstrapped mediations (5000 resamples) were run, with perceived weight stigma (POTS) as the predictor, the internalised weight stigma (WBIS) as the mediator, and each of the psychosocial variables listed above as the outcome variables. In all these analyses, we controlled for BMI.

#### Correction to Reduce the Risk of Type I Error

The large number of mediation tests (i.e. 51) raise concerns about the probability of Type I errors. There is much debate about family-wise error rate, the types of corrections available, and the appropriateness and effectiveness of such corrections [42–45]. This is partly due to the difficulty of estimating the degree of dependence between the different tests conducted on the same data set. Based on a reviewer’s recommendation, we implemented the Hochberg procedure (see Cao and Zhang) [46]. We did this after classifying the variables into relevant conceptual domains (i.e. disordered eating, psychological quality of life, physical quality of life and health, body image, and psychological distress/functioning variables). A complete list of this procedure being applied to these different domains in our data can be found in Table S2.

## Results

Table 2 shows descriptive statistics for the demographics of the current sample. Due to the large number of measures in the current study, means, standard deviations, and internal consistency scores for all non-demographic measures used are presented in Table S1. In sum, perceived weight stigma was positively associated with negative correlates such as disordered eating, symptoms of depression and anxiety, stress, and pain except for EDE-Q restraint, DEBQ restraint, DEBQ emotional eating, QEWP, WEL restraint, AQoL pain, and several MBSRQ subscales. Perceived weight stigma was also negatively associated with positive correlates, such as quality of life, motivation to exercise, and self-esteem (see Table S3 for full list of correlations of all psychosocial and physical correlate variables with perceived and internalised weight stigma, and BMI). Similar relationships were found between internalised weight stigma and these biopsychosocial correlates.

### Mediations

**Fig. 1:**
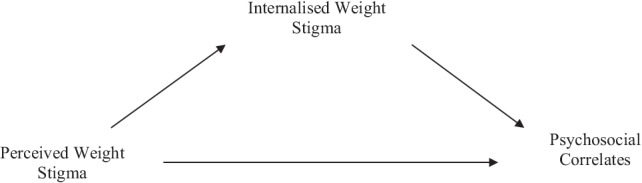
Internalised weight stigma as a mediator of the relationship between perceived weight stigma and biopsychosocial correlates

Mediation analyses were conducted to estimate the indirect effect of perceived weight stigma on psychosocial correlates via internalised weight stigma, after controlling for BMI (see Fig. 1). Correlates were separated into three conceptual domains: eating behaviour, quality of life and physical health, and other psychosocial health correlates.

Tables 3, 4, and 5 present the mediation analyses results for each of these domains. The tables report the unstandardised and standardised effect size estimates and standard errors for the total effect (*c*), direct effect (*cʹ*), the individual predictor-mediator (*a*) and mediator-outcome (*b*) pathways, and the indirect effect (a*b) with confidence intervals and its corresponding *p* value. Importantly, bolded variables in Tables 3, 4, and 5 indicate the mediation was significant after correcting to reduce the risk of Type I error with the Hochberg procedure. Note that some measures had confidence intervals that did not include zero (e.g. EDEQ-R, QEWP), indicating an effect; however, these *p* values did not meet the threshold for significance after the Hochberg procedure.

**Table 3:** Unstandardised and standardised regression coefficients for the total effects, each pathway of the mediation, and the indirect effect for each eating behaviour correlate (all analyses control for BMI)

Psychosocial Correlates	*c* (SE)	*β c*	*c’* (SE)	*β c’*	*a* (SE)	*β a*	*b* (SE)	*β b*	a*b (SE)	95% CIs	β a*b	a*b p
EDE-Q (global)	0.293 (0.072)	0.285***	0.160 (0.074)	0.156*	0.329 (0.083)	0.272***	0.401 (0.051)	0.473***	**0.132 (0.037)**	**0.064, 0.212**	**0.129**	** < .001**
Restraint	0.143 (0.102)	0.104	0.083 (0.111)	0.061	0.329 (0.084)	0.272***	0.180 (0.077)	0.160*	0.059 (0.030)	0.012, 0.135	0.043*	.049
Eating concern	0.343 (0.121)	0.202**	0.256 (0.151)	0.151	0.329 (0.086)	0.272***	0.263 (0.097)	0.187**	0.086 (0.040)	0.022, 0.182	0.051*	.032
Shape concern	0.289 (0.079)	0.262***	0.129 (0.071)	0.117	0.329 (0.087)	0.272***	0.486 (0.063)	0.533***	**0.160 (0.048)**	**0.071, 0.265**	0.145***	**.001**
Weight concern	0.395 (0.091)	0.308***	0.172 (0.073)	0.134*	0.329 (0.085)	0.272***	0.674 (0.058)	0.636***	**0.222 (0.061)**	**0.104, 0.346**	0.173***	** < .001**
Dutch Eating Behaviour Questionnaire												
Restraint	− 0.003 (0.044)	− 0.006	− 0.020 (0.055)	− 0.034	0.330 (0.087)	0.273***	0.049 (0.037)	0.101	0.016 (0.013)	− 0.005, 0.047	0.027	.229
Emotional eating	0.078 (0.070)	0.082	− 0.045 (0.073)	− 0.048	0.330 (0.086)	0.273***	0.374 (0.053)	0.479***	**0.123 (0.036)**	**0.059, 0.203**	0.131***	** < .001**
External eating	0.16 (0.052)	0.221**	0.136 (0.054)	0.189*	0.329 (0.086)	0.272***	0.070 (0.046)	0.117	0.023 (0.017)	− 0.005, 0.066	0.032	.195
Three-Factor Eating Questionnaire												
Cognitive restraint	− 0.638 (0.285)	− 0.165*	− 0.755 (0.279)	− 0.195**	0.329 (0.085)	0.272***	0.355 (0.223)	0.111	0.117 (0.081)	− 0.015, 0.317	0.030	.149
Disinhibition	0.529 (0.246)	0.158*	0.160 (0.230)	0.048	0.329 (0.086)	0.272***	1.121 (0.197)	0.406***	**0.369 (0.119)**	**0.172, 0.661**	0.111**	**.002**
Hunger	0.956 (0.28)	0.244***	0.769 (0.285)	0.196**	0.329 (0.087)	0.272***	0.568 (0.249)	0.176*	0.187 (0.102)	0.027, 0.437	0.047	.053
Clinical Impairment Assessment	3.316 (0.84)	0.284***	1.301 (0.705)	0.111	0.323 (0.088)	0.267***	6.237 (0.540)	0.647***	**2.015 (0.566)**	**0.906, 3.128**	0.173***	** < .001**
Questionnaire on Eating and Weight Patterns	1.084 (0.619)	0.129	0.500 (0.626)	0.059	0.329 (0.087)	0.272***	1.771 (0.504)	0.255***	0.584 (0.242)	0.204, 1.181	0.069*	.016
Weight-Efficacy Lifestyle Questionnaire (Restraint)	− 0.222 (0.118)	− 0.140	− 0.122 (0.112)	− 0.077	0.321 (0.086)	0.264***	− 0.311 (0.102)	− 0.238**	− 0.100 (0.047)	− 0.215, -0.026	− 0.062*	.036

*Note. c* = total effect, *c’* = direct effect, *a* = predictor-mediator relationship, *b* = mediator-outcome relationship, a*b = indirect effect. ***p < .001, **p < .01, *p < .05. Exact *p* values are reported for all standardised indirect effects. Bolded variables are significant mediations after adjusting for Type I error

**Table 4:** Unstandardised and standardised regression coefficients for the total effects, each pathway of the mediation, and the indirect effect for each quality of life correlate and pain (all analyses control for BMI)

Psychosocial correlates	*c* (SE)	*β* c	*c’* (SE)	*β* c’	*a* (SE)	*β* a	*b* (SE)	*β* b	a*b (SE)	95% CIs	*β* a*b	a*b p
Assessment of QoL (total)	− 0.021 (0.009)	− 0.157*	− 0.007 (0.011)	− 0.054	0.300 (0.085)	0.252***	− 0.046 (0.009)	− 0.411***	− **0.013 (0.004)**	− **0.024, -0.006**	− 0.103**	**.003**
AQoL mental health	− 0.028 (0.011)	− 0.181*	− 0.004 (0.012)	− 0.031	0.319 (0.088)	0.263***	− 0.073 (0.009)	− 0.568***	− **0.023 (0.006)**	− **0.037, -0.009**	− 0.149***	** < .001**
Happiness	− 0.023 (0.011)	− 0.152*	− 0.007 (0.011)	− 0.050	0.319 (0.085)	0.263***	− 0.049 (0.008)	− 0.387***	− **0.015 (0.004)**	− **0.026, -0.007**	− 0.102***	**.001**
Mental health	− 0.019 (0.008)	− 0.165*	− 0.004 (0.009)	− 0.038	0.329 (0.086)	0.272***	− 0.046 (0.007)	− 0.467***	− **0.015 (0.004)**	− **0.024, -0.007**	− 0.127***	** < .001**
Coping	− 0.029 (0.010)	− 0.211**	− 0.015 (0.011)	− 0.111	0.330 (0.085)	0.272***	− 0.042 (0.008)	− 0.372***	− **0.014 (0.004)**	− **0.024, -0.006**	− 0.101**	**.003**
Relationships	− 0.035 (0.01)	− 0.245***	− 0.016 (0.01)	− 0.111	0.329 (0.085)	0.272***	− 0.059 (0.008)	− 0.495***	− **0.019 (0.005)**	− **0.032, -0.009**	− 0.135***	**.001**
Self-worth	− 0.046 (0.013)	− 0.246***	− 0.015 (0.012)	− 0.081	0.330 (0.087)	0.272***	− 0.094 (0.009)	− 0.606***	− **0.031 (0.008)**	− **0.047, -0.015**	− 0.165***	** < .001**
AQoL physical health	− 0.027 (0.015)	− 0.131	− 0.026 (0.016)	− 0.127	0.299 (0.086)	0.251***	− 0.001 (0.013)	− 0.009	− 0.005 (0.004)	− 0.009, 0.007	− 0.002	.904
Independent living	− 0.019 (0.01)	− 0.123	− 0.020 (0.011)	− 0.129	0.329 (0.085)	0.272***	0.002 (0.009)	0.021	0.006 (0.003)	− 0.005, 0.007	0.005	.779
Pain	− 0.025 (0.018)	− 0.103	− 0.024 (0.021)	− 0.096	0.299 (0.085)	0.251***	− 0.005 (0.016)	− 0.024	− 0.001 (0.004)	− 0.012, 0.008	− 0.006	.751
Senses	− 0.013 (0.009)	− 0.111	− 0.012 (0.009)	− 0.102	0.329 (0.085)	0.272***	− 0.003 (0.007)	− 0.032	− 0.001 (0.002)	− 0.006, 0.003	− 0.008	.686
Impact of Weight QoL (total)	7.807 (1.585)	0.329***	4.805 (1.539)	0.202**	0.329 (0.086)	0.272***	9.101 (1.123)	0.464***	**3.002 (0.903)**	**1.425, 4.928**	0.126***	** < .001**
Physical function	1.581 (0.703)	0.156*	1.336 (0.786)	0.131	0.329 (0.086)	0.271***	0.741 (0.614)	0.088	0.243 (0.227)	− 0.111, 0.799	0.024	.284
Self-esteem	2.021 (0.483)	0.299***	0.622 (0.353)	0.092	0.329 (0.087)	0.271***	4.247 (0.273)	0.763***	**1.398 (0.388)**	**0.661, 2.189**	0.207***	** < .001**
Sexual life	0.614 (0.404)	0.114	− 0.073 (0.391)	− 0.013	0.331 (0.088)	0.274***	2.073 (0.279)	0.466***	**0.687 (0.207)**	**0.317, 1.138**	0.128***	** < .001**
Public distress	2.426 (0.301)	0.446***	2.056 (0.346)	0.378***	0.329 (0.086)	0.272***	1.122 (0.241)	0.249***	**0.370 (0.128)**	**0.159, 0.664**	0.068**	**.004**
Work	1.265 (0.286)	0.313***	0.965 (0.327)	0.239**	0.317 (0.089)	0.261***	0.945 (0.235)	0.284***	**0.300 (0.122)**	**0.107, 0.594**	0.074*	**.014**
Brief Pain Inventory												
Pain interference	0.314 (0.208)	0.116	0.266 (0.224)	0.099	0.401 (0.081)	0.332***	0.120 (0.181)	0.053	0.048 (0.074)	− 0.089, 0.212	0.017	.519
Pain severity	0.319 (0.165)	0.145	0.362 (0.175)	0.165*	0.368 (0.086)	0.295***	− 0.116 (0.138)	− 0.066	− 0.042 (0.053)	− 0.164, 0.052	− 0.019	.427

*Note*. *c* = total effect, *c’* = direct effect, *a* = predictor-mediator relationship, *b* = mediator-outcome relationship, a*b = indirect effect. ***p < .001, **p < .01, *p < .05. Exact *p* values are reported for all standardised indirect effects. Bolded variables are significant mediations after adjusting for Type I error

**Table 5:** Unstandardised and standardised regression coefficients for the total effects, each mediation pathway, and indirect effect for anxiety, depression, body image, and other biopsychosocial correlates (all analyses control for BMI)

Psychosocial correlates	*c* (SE)	*β* c	*c’* (SE)	*β* c’	*a* (SE)	*β* a	*b* (SE)	*β* b	a*b (SE)	95% CIs	*β* a*b	a*b p
Depression Anxiety Stress Scale												
Anxiety	2.648 (0.563)	0.333***	1.914 (0.575)	0.241***	0.329 (0.084)	0.272***	2.224 (0.428)	0.339***	**0.733 (0.245)**	**0.324, 1.299**	0.092**	**.003**
Stress	1.505 (0.700)	0.158*	0.412 (0.747)	0.043	0.329 (0.085)	0.272***	3.314 (0.534)	0.421***	**1.093 (0.343)**	**0.485, 1.825**	0.114***	**.001**
Depression	2.851 (0.736)	0.279***	1.499 (0.802)	0.147	0.329 (0.087)	0.272***	4.098 (0.571)	0.486***	**1.352 (0.391)**	**0.651, 2.174**	0.132***	** < .001**
Patient Health Questionnaire	0.668 (0.242)	0.202**	0.111 (0.253)	0.033	0.245 (0.056)	0.353***	2.265 (0.370)	0.476***	**0.556 (0.162)**	**0.275, 0.920**	0.168***	** < .001**
GAD Assmt	1.259 (0.321)	0.286***	0.839 (0.364)	0.191*	0.339 (0.086)	0.283***	1.233 (0.259)	0.336***	**0.419 (0.139)**	**0.184, 0.738**	0.095**	**.003**
Tolerance of Mood Scale	0.129 (0.033)	0.276***	0.067 (0.034)	0.144*	0.326 (0.087)	0.270***	0.188 (0.025)	0.486***	**0.061 (0.018)**	**0.027, 0.099**	0.131***	**.001**
Rosenberg Self-Esteem Scale	− 1.526 (0.436)	− 0.253***	− 0.524 (0.408)	− 0.087	0.329 (0.086)	0.272***	− 3.035 (0.283)	− 0.611***	− **1.001 (0.271)**	− **1.560,** − **0.484**	− 0.166***	** < .001**
Exercise-Avoidance Motivation Scl	0.574 (0.101)	0.389***	0.359 (0.088)	0.243***	0.329 (0.086)	0.272***	0.652 (0.083)	0.534***	**0.215 (0.061)**	**0.103, 0.346**	0.145***	** < .001**
Multi-dimensional Body-Self Relations Questionnaire												
Appearance evaluation	− 0.081 (0.044)	− 0.133	0.002 (0.046)	0.004	0.329 (0.085)	0.272***	− 0.255 (0.032)	− 0.508***	− **0.084 (0.025)**	− **0.139,** − **0.040**	− 0.138***	** < .001**
Appearance orientation	− 0.047 (0.041)	− 0.082	− 0.096 (0.044)	− 0.167*	0.329 (0.086)	0.272***	0.149 (0.032)	0.312***	**0.049 (0.017)**	**0.020, 0.090**	0.085**	**.006**
Fitness evaluation	− 0.099 (0.063)	− 0.117	− 0.048 (0.068)	− 0.056	0.329 (0.085)	0.272***	− 0.156 (0.05)	− 0.222**	− 0.051 (0.021)	− 0.106, − 0.017	− 0.061*	.019
Fitness orientation	− 0.008 (0.045)	− 0.013	0.007 (0.046)	0.011	0.329 (0.088)	0.272***	− 0.047 (0.037)	− 0.091	− 0.015 (0.013)	− 0.047, 0.006	− 0.024	.255
Health evaluation	− 0.071 (0.048)	− 0.106	− 0.011 (0.048)	− 0.015	0.329 (0.086)	0.272***	− 0.184 (0.037)	− 0.332***	− **0.061 (0.021)**	− **0.110,** − **0.027**	− 0.091**	**.004**
Health orientation	− 0.138 (0.041)	− 0.237***	− 0.102 (0.041)	− 0.174*	0.329 (0.086)	0.272***	− 0.111 (0.032)	− 0.230***	− 0.036 (0.014)	− 0.071, − 0.014	− 0.062*	.011
Illness orientation	− 0.101 (0.056)	− 0.132	− 0.104 (0.054)	− 0.137	0.329 (0.086)	0.272***	0.010 (0.047)	0.016	0.003 (0.015)	− 0.028, 0.036	0.004	.836
Body areas satisfaction	− 0.092 (0.036)	− 0.186**	− 0.031 (0.029)	− 0.063	0.329 (0.086)	0.272***	− 0.186 (0.027)	− 0.454***	− **0.061 (0.018)**	− **0.103,** − **0.028**	− 0.123***	**.001**
Overweight preoccupation	− 0.038 (0.051)	− 0.055	− 0.099 (0.049)	− 0.145*	0.329 (0.087)	0.272***	0.186 (0.038)	0.328***	**0.061 (0.021)**	**0.029, 0.109**	0.089**	**.003**
Self-classified weight	0.047 (0.029)	0.112	0.017 (0.026)	0.041	0.329 (0.085)	0.272***	0.089 (0.025)	0.259***	0.029 (0.012)	0.010, 0.058	0.071*	.015

*Note. c* = total effect, *c’* = direct effect, *a* = predictor-mediator relationship, *b* = mediator-outcome relationship, a*b = indirect effect. ***p < .001, **p < .01, *p < .05. Exact *p* values are reported for all standardised indirect effects. GAD Assmt = Generalised Anxiety Disorder Assessment; MBSRQ scores – low scores indicate: AE = negative body image, dissatisfied with appearance, AO = apathetic about appearance, looks viewed as not particularly important, FE = considers self unfit, does not value physical fitness nor regularly exercises, FO = does not value physical fitness nor regularly exercises, HE = feels unhealthy and ill, HO = apathetic about health, IO = not very alert to personal symptoms of physical illness, BAS = unhappy with size/appearance, OP = minimal feelings of fat anxiety, dieting/restraint, and weight vigilance, WC = low self-perceived weight. Bolded variables are significant mediations after adjusting for Type I error

#### Disordered Eating Variables

Internalised weight stigma mediated the relationship between perceived weight stigma and some disordered eating measures (i.e. 6 of 14 measures were significant after correcting to reduce the risk of Type I error, including measures of emotional eating, eating-specific psychosocial impairment, and shape and weight concern). One measure of disinhibition was significant and one was not significant. All measures of dietary restriction/restraint were non-significant, as were external eating, hunger, and eating concern (see Table 3).

#### Quality of Life and Physical Health Variables

Internalised weight stigma mediated the relationship between perceived weight stigma and all measures of psychological quality of life, but *not* physical quality of life or pain (see Table 4).

#### Other Psychosocial Variables

Internalised weight stigma mediated the relationship between perceived weight stigma and symptoms of depression and anxiety, exercise avoidance, maladaptive responses to intense negative mood states (general and eating specific), self-esteem, and 5 of 10 body image subscales (see Table 5).

Across all analyses, all significant relationships were in the expected direction. Specifically, higher perceived weight stigma was associated with higher internalised weight stigma, which was in turn associated with more adverse scores in the correlates (i.e. higher symptoms of anxiety, and lower psychological quality of life).

## Discussion

The current study aimed to (a) assess the bivariate relationship between perceived and internalised weight stigma and psychosocial and physical correlates in individuals seeking bariatric surgery, and (b) estimate the mediating role of internalised weight stigma on the relationships between perceived stigma and said correlates [14]. All mediations were corrected to reduce the risk of Type I error. As expected, perceived and internalised weight stigma were significantly, positively correlated. Furthermore, both types of stigma were significantly associated with negative correlates (e.g. higher disordered eating and symptoms of depression, and lower quality of life), with a few exceptions.

We also found statistical evidence of the mediating role of internalised weight stigma on the relationship between perceived weight stigma and several psychological correlates, after controlling for BMI. These mediations were all in the expected direction, where higher perceived stigma is associated with higher internalised stigma, which in turn is associated with more negative outcomes. However, several estimated mediations were not significant for (a) some measures of disordered eating, including external eating and all measures of restraint, (b) some measures of body image, and (c) all measures of physical health, specifically, physical quality of life and pain.

Our mediation findings are consistent with those in other populations (e.g. university students, community and clinical participants), where internalised weight stigma was found to mediate the relationship between perceived weight stigma and psychosocial correlates, after controlling for BMI [47–52]. Moreover, our findings extend previous research conducted with individuals seeking bariatric surgery, by assessing a number of new psychosocial correlates beyond depression and anxiety [17]. Our study is the first to estimate this mediation effect in other correlates, such quality of life and disordered eating.

Though cross-sectional, our findings provide partial support to Tylka et al.’s [14] model of the role of internalised weight stigma. Specifically, internalised weight stigma mediated the relationship between perceived weight stigma and psychological well-being, as indicated above. However, internalised weight stigma did not mediate the relationship between perceived weight stigma and physical health correlates (i.e. pain and physical quality of life) and some disordered eating and body image measures, at least in this sample of individuals seeking bariatric surgery.

Research suggests weight stigma is linked to several poor physiological health correlates [53] and may partially explain the relationship between BMI and physiological health [54]. However, the physical quality of life measures used in this study make specific reference to issues associated with mobility and/or environmental barriers. Items from the AQoL, for example, ask: ‘How much help do you need with jobs around the house (e.g., preparing food, cleaning the house or gardening)?’ and ‘Thinking about how easy or difficult it is for you to get around by yourself outside your house (e.g., shopping, visiting)’. Variance in these types of correlates may be better explained by other more physical factors (e.g. ability, fitness, or weight) instead of weight stigma. Table S3 shows that in our sample, BMI was itself significantly negatively correlated with three of four physical quality of life measures across two subscales (senses subscale non-significant).

We also found non-significant findings for the pain correlates. Interestingly, Table S3 shows the observed correlation between BMI and pain was significant and positive for the pain severity subscale, but non-significant for pain interference. Therefore, it is important to distinguish between physical quality of life and pain on one hand, and physiological health and physical impairment on the other, as we found weight stigma is not associated with the former but a substantial amount of previous research has found weight stigma is associated with the latter.

### Disordered Eating Correlates

We had mixed findings on disordered eating correlates. Specifically, internalised weight stigma did not mediate the relationship between perceived weight stigma and dietary restriction/restraint after correcting to reduce the risk of Type I error. Interestingly, although a systematic review found disordered eating correlates have been the most studied measure for this mechanism in the literature [16], restraint was measured only in three of the eight studies focusing on disordered eating. Two of these studies reported significant mediation for restraint, but this research was not conducted with bariatric surgery samples. The evidence for dietary restriction/restraint as a correlate of weight stigma is not only less well known than other measures of disordered eating but also inconclusive at present. Thus, additional studies looking at the present mediation pathway for restraint will be needed to estimate the size of the effect (or to determine if it is indeed zero).

As mentioned above, internalised weight stigma did not mediate the relationship between perceived weight stigma and external eating, eating concern, and hunger. We could see no obvious explanation for these non-significant findings. However, the difference in significant and non-significant disordered eating findings may have to do with the relative strength of emotion associated with each type of disordered eating. Specifically, it may be that restraint and external eating (i.e. intentions or thoughts about eating) are less distressing or pronounced for individuals than emotional eating and disinhibition, which are often accompanied by strong feelings of distress (as are shape and weight concern; see Table 3). Previous research has shown that both emotional eating and disinhibition are positively associated with distress [55, 56]. This may in part explain why we found significant indirect effects for the latter measures (with one exception) but not the former. Lastly, we could find no obvious explanation for the significant versus non-significant findings in the body image subscales.

### Estimating Effect Size in Mediation Models

Reporting appropriate and meaningful estimates of effect sizes for mediation is a complex topic [57, 58]. As Wen and Fan [57] state, ‘*no single index appears to be a viable mediation effect size measure*’ (p. 199). They recommend reporting both the standardised and unstandardised total, and direct and indirect effects to estimate the effect sizes of the mediation, which we have done here. In the current study, many of the standardised effect size estimates for the indirect pathways we found for psychosocial correlates (i.e. the expected mediations) appear small (most were < 0.20). However, these expected mediations had a strong theoretical explanation, and these estimates were not trivial, with a few exceptions. Physical health measures (i.e. the exploratory mediations) were all non-significant, and in these cases, effect sizes were miniscule and were not close to significance. Thus, we can be somewhat more confident that these estimated mediations are genuine effects given the consistency of the findings.

### Limitations of the Current Study and Future Research

Given the cross-sectional nature of the evidence presented here, our findings provide *necessary* but not *sufficient* evidence to support the causal pathways in the model. Future research should consider conducting longitudinal studies on the mediating role of internalised weight stigma to further clarify this mechanism, as experimental studies in this domain (i.e. exposing individuals to experiences of weight stigma) pose several ethical issues. Future research should also consider the complexities in the possible bi-directional relationships between the different types of weight stigma. For example, it might be useful to have clarity on whether individuals high in internalised weight stigma are more sensitive to, or have a lower threshold for, experiences and perceptions of weight stigma that occur in the environment.

Our findings must be interpreted in the context of the psychometric limitations of the Weight Bias Internalisation Scale [59]. This scale has demonstrated adequate to good psychometric properties in the current study, previous research with samples of community participants [60], and individuals seeking bariatric surgery [13]. It is also the most frequently used measure of internalised weight stigma in the literature, making it comparable to previous research. However, some recent evidence indicates that this scale may be confounded by other known predictors of disordered eating, such as body image and self-esteem (see Meadows and Higgs) [61]. Although this consideration is outside the scope of the current paper, a discussion of this issue can be found elsewhere [16, 62, 63].

### Implications and Conclusion

As we have made clear elsewhere [16], ours and others’ suggestion to address the internalisation of weight stigma does in no way imply that the target of stigma is at fault or to blame for having internalised it. Indeed, internalisation of weight stigma, like internalisation of the thin ideal, would not exist in a world in which weight stigma was not the norm. We strongly advocate for the elimination of weight stigma in society. However, whilst cultural and societal changes are being enacted to eliminate weight stigma (which is likely to take many years), breaking the link between perceived and internalised weight stigma may help mitigate negative correlates in the shorter term. There is some evidence of interventions that may successfully decrease internalised weight stigma [64, 65].

Our results add support to previous findings highlighting the importance of including psychological components as part of a multi-disciplinary approach to clients’ care both before and after bariatric surgery [66, 67]. The fact that the relationships reported in this paper are present after controlling for BMI suggests that some of the negative correlates observed could be maintained even in the presence of surgical weight loss. Thus, interventions that address internalised weight stigma may improve patient outcomes beyond weight loss alone, and could be implemented by healthcare professionals as part of long-term care [64, 68, 69]. Such interventions would likely include psychoeducation about weight science and its complexities, challenging oversimplifications about weight and health, and cognitive restructuring and reappraisal, amongst many other things.

## Supplementary Information

Below is the link to the electronic supplementary material.

Supplementary file1 (DOCX 65 kb)
