# Supplementary material for: Internalised Weight Stigma Mediates Relationships Between Perceived Weight Stigma and Psychosocial Correlates in Individuals Seeking Bariatric Surgery: a Cross-sectional Study
Source: Obes Surg. 2022 Sep 12;32(11):3675–86. doi: 10.1007/s11695-022-06245-z (PMC9613718; doi:10.1007/s11695-022-06245-z)
Supplement: Supplementary file 1 — Supplementary file1 (DOCX 65 kb) [file 11695_2022_6245_MOESM1_ESM.docx]

**Internalised Weight Stigma Mediates Relationships Between Perceived Weight Stigma and Psychosocial Correlates in Individuals Seeking Bariatric Surgery: A Cross-Sectional Study**

Shortened Title: Weight Stigma Correlates

**Keywords:** internalised weight stigma, mediation, bariatric surgery, disordered eating, quality of life, depression, anxiety.

**Funding:** No conflicts or competing interests to declare.

**Measures**

***Weight Stigma***

**Perception of Teasing Scale (POTS; General Weight Teasing Subscale) (1).** Data collected only included the 6-item general weight teasing subscale of the POTS (i.e., items 1-6; original scale has 11 items). These items ask participants about their perceptions and experiences of weight stigma in terms of (a) the frequency of events and (b) how upset they were by these events (hence every item has ‘a’ and ‘b’). For both frequency and effect, items are measured on a 5-point Likert scale from (1) never to (5) very often for frequency items, and (1) not upset to (5) very upset for the effect items, respectively. Scores were calculated both as the mean of the frequency of the events (i.e., ‘a’ items), and as the mean of how upset participants were by the events (i.e., ‘b’ responses)^[[1]](#footnote-1)^. Sample items include: “People called you names like ‘fatso’” and “People pointed at you because you are overweight”. High scores indicate high levels of (a) frequency of events of teasing, and (b) being negatively effected by weight-based teasing. Mediations in the current study were run on the frequency subscale, but the results of the upset subscale can be found here in supplementary material, in Table S3-S5.

**Weight Bias Internalisation Scale Modified (WBIS-11) (2, 3).** This 11-item scale measures the extent to which participants internalise weight stigma (i.e., the extent to which individuals devalue themselves as a result of negative stereotypes about weight). Sample items include: “I am less attractive than most other people because of my weight” and “My weight is a major was that I judge my value as a person”. Items are rated on 7-point Likert scale (1 = strongly disagree; 7 = strongly agree). High scores indicate high levels of weight bias internalisation.

**Experienced Weight Stigma (EWS).** This 3-item scale was developed ad-hoc by those who compiled the measures of the original study. Sample items include asking participants “how often are you: (1) teased about being overweight/fat (2) discriminated against because of your being overweight/fat, and (3) bullied (verbal abuse, harassment) because of being overweight/fat”. Responses are measured on a 5-point Likert scale ranging from (1) never to (5) at least once a week. High scores indicate high levels of experienced weight stigma. This scale was not used in the current analysis (see below).

***Eating and/or Exercise Behaviour***

**Clinical Impairment Assessment (CIA) (4)**. This 16-item scale measures psychosocial impairment related to disordered eating. The scale begins with the following preface, which is to be applied to all 16-item listed: “Over the past 28 days, to what extent have your eating habits or exercising or feelings about your eating, shape, or weight…”. Sample items include: “… made it difficult to concentrate” and “… made you feel guilty”. Items are measured on a 4-point Likert scale from (0) not at all to (3) a lot. High scores indicate higher psychosocial impairment.

**Dutch Eating Behaviour Questionnaire (DEBQ) (5).** This 33-item scale measures eating behaviour via three subscales: restrained, emotional, and external eating. Sample items from each subscale, respectively, include: “Do you watch exactly what you eat?” “Do you have the desire to eat when you depressed or discouraged?” and “If food smells and looks good to you do you eat more than usual?”. Items are measured on a 5-point Likert scale from (1) never to (5) very often. High scores indicate higher levels of disordered eating behaviours.

**Eating Disorder Examination Questionnaire (EDE-Q) (6, 7).** This 30-item scale measures disordered eating behaviour in the last 28 days. The scale has a global score and four subscales, measuring restraint, eating concern, weight concern, and shape concern. Sample items from the restraint and weight concern subscales, respectively, include: “Have you tried to follow definite rules regarding your eating (for example, calorie limit) in order to influence your shape or weight (whether or not you have succeeded)?” and “Have you had a strong desire to lose weight?”. Items are rated on a 7-point Likert scale from (0) no days to (6) every day. High scores indicate higher levels of disordered eating.

**Exercise-Avoidance Motivation Scale (EAMS) (8).** This 8-item scale measures exercise avoidance and motivation by describing negative situations that individuals with higher weight may experience. Sample items include: “I avoid going to the gym when I know there will be a lot of thin people there” and “I avoid going out in public places because I am afraid that people will make comments about my size”. Items are measured on a 7-point Likert scale from (1) not true at all to (7) completely true. High scores indicate more frequent negative experiences due to their weight.

**The Questionnaire on Eating and Weight Patterns (QEWP-R) (9).** This 18-item scale measures participants’ history and symptoms of disordered eating over the past three months, focussing primarily on symptoms of binge eating disorder. Sample items include: “During the past three months, did you often eat or nibble continuously (‘graze’) over an extended period of time?”. Items are rated either on a 5-point Likert scale from 1 (less than once a week) to 5 (nearly every day) or dichotomously (yes/no). High scores indicate greater disordered eating symptoms.

**Three Factor Eating Questionnaire (TFEQ) (10).** This 51-item scale measures eating behaviour in three separate subscales: restrained eating, disinhibition of control of eating, and perceived hunger. Sample items from each subscale, respectively, include: “When I have eaten my quota of calories, I am usually good about not eating any more” “I usually eat too much at social occasions, like parties and picnics” and “I am usually so hungry that I eat more than three times a day”. Scores are considered in isolation (i.e., high scores on restraint indicate higher restrained eating).

**Weight Efficacy Lifestyle Questionnaire (WEL) (11).** This 20-item scale measures participant’s self-reported ability (or self-efficacy) to resist eating in certain circumstances. The measure has five subscales of situational factors: negative emotions, availability, social pressure, physical discomfort, and positive activities. A sample item from this scale is: “I can resist eating even when I am at a party”. Items are rated according to the individuals’ feelings of confidence to eat or not eat food on a 10-point Likert scale from 0 “Not confident” to 9 “Very confident”. High scores indicate high self-efficacy in being able to exercise restraint.

***Quality of Life***

**Assessment of Quality of Life (AQoL) (12).** This 35-item scale measures general quality of life across eight dimensions, which all fall under one of two umbrella dimensions: physical (independent living, pain, and senses) or psychosocial (happiness, mental health, coping, relationships, self-worth) quality of life. A sample item from the happiness dimension is: “How content are you with your life?”. Items are rated on a 5-point Likert scale. Possible responses change from item to item (e.g., some items as from “never” to “all the time”, others “not at all” to “extremely”. High scores indicate good quality of life.

**Impact of Weight on Quality of Life-Lite (IWQOL-Lite) (13).** This 31-item scale measures quality of life in the context of weight concerns across five subscales: physical function, self-esteem, sexual life, public distress, and work. Sample items include: “Because of my weight I have trouble picking up objects” and “Because of my weight I am less productive than I could be”. Items are rated on 5-point Likert scale from 1 “never true” to 5 “always true”. High scores indicate poorer quality of life.

***Depression, Anxiety, and Stress***

**Depression Anxiety Stress Scales (DASS-21) (14).** This 21-item scale measures negative emotional states on three subscales: depression, anxiety, and stress. Sample items from each include: “I couldn’t seem to experience any positive feeling at all”, “I was aware of dryness of my mouth”, and “I found it hard to wind down”, respectively. Items are rated on a 4-point Likert scale from 0 “did not apply to me at all” to 3 “applied to me very much, or most of the time” over the past week. High scores indicate higher levels of negative emotional states.

**The Generalized Anxiety Disorder Scale (GAD-7) (15).** This 7-item scale measures the presence of anxiety symptoms. Each item begins with the premise “Over the last two weeks, how often have you been bothered by the following problems”. A sample item is: “Feeling nervous, anxious, or on edge”. Items are rated on a 4-point Likert scale from 0 “not at all” to 3 “nearly every day”. High scores indicate higher levels of anxiety severity.

**Patient Health Questionnaire (PHQ) (16).** This 9-item scale measures the presence and severity of depressive symptoms. Each item begins with the premise “Over the last two weeks, how often have you been bothered by any of the following”. Sample items include: “Poor appetite or overeating” and “little interest of pleasure in doing things”. Items are rated on a 4-point Likert scale from 0 “not at all” to 3 “nearly every day”. High scores indicate higher levels of depressive symptoms.

***Other Psychosocial Outcomes and Pain***

**The Multidimensional Body-Self Relations Questionnaire (MBSRQ) (17).** This 69-item scale measures affective, behavioural, and cognitive components of body image across 10 subscales (appearance evaluation, appearance orientation, fitness evaluation, fitness orientation, health evaluation, health orientation, illness orientation, body areas satisfaction, overweight preoccupation, self-classified overweight). A sample item from the appearance evaluation subscale is: “I am physically unattractive”. Items are rated on a 5-point Likert scale, from 1 “Definitely disagree” to 5 “Definitely agree”. High scores on the appearance evaluation subscale, for example, indicate positive body image and satisfaction with appearance.

**Rosenberg Self-Esteem Scale (RSE) (18).** This 10-item scale measures self-acceptance and self-worth, together forming global self-esteem. A sample item from this scale is: “I feel that I have a number of good qualities”. Items are rated on a 4-point Likert scale; from 1 “Strongly agree” to 4 “Strongly disagree”. High scores indicate high self-esteem.

**Tolerance of Mood States Scale (TOMS) (19).** This measure consists of two separate scales. First, is a 23-item scale (with three subscales: negative affect, anger, and anxiety) that measures participant’s self-reported tolerance of mood states. In this scale, participants are asked to rate how they feel about several emotions, such as being lonely, sad, or irritated from 1 “I really like this feeling and am always pleased to expierence it” to 6 “I can not stand this feeling and try to avoid it whenever possible”. The scale development paper found this first scale was not valid. Thus, though data was collected on it, this first scale was not included in current study. Second is an 11-item scale measuring coping behaviors, both general and eating-specific, in response to strong emotions (e.g., “eat something/eat more than usual” and “be irritable with others”). Items on this scale are rated from 1 “never” to 5 “always”. High scores indicate a greater dislike for negative mood states in the first scale and a higher likelihood of engaging in adverse behaviours to cope in the second scale.

**Brief Pain Inventory (BPI) (20).** This 9-item scale measures the severity of pain and how pain interferes with daily life. A sample item from the pain severity subscale is “Please rate your pain by circling the one number that best describes your pain on average”. Items are rated on an 11-point Likert scale from 0 “No pain” to 10 “Pain as bad as you can imagine”. High scores indicate higher pain severity and higher pain inference in daily life.

Note that a scale was created ad-hoc by the researchers in the original study, which aimed to measure experienced weight stigma (describe above). As data was already collected on the POTS, this measure was not needed for our analyses. This scale was not included in planning and running the data analysis.

**Data Cleaning and Screening**

Cases were first screened for missing values. Three cases had more than 50% missing data and were excluded from further analysis. Twelve cases had more than 5% missing data. We only excluded these cases in analyses for which they had missing data. For the whole sample, the POTS and the BPI-P and BPI-I had more than 5% missing data (5.6%, 6.5%, and 10.2%, respectively). A missing values analysis revealed that 5 of 51 measures (DEBQ external eating, IWQoL distress, DASS (A, D), EAMS) were significantly higher and 2 of 51 (AQoL coping, MBSRQ AE) were significantly lower for those giving a response on the POTS vs. not.

We also conducted a missing values analysis on the BPI-P and BPI-I. We found significant differences between reporters vs. non-reporters on BPI-Pain Severity and: MBSRQ IO, TFEQ CR, AQoL Physical Dimension, and DEBQ Ext (higher on the first three, lower on last outcome). In addition, we found significant differences between reporters vs. non-reporters on BPI-Interference and: IWQoL total, IWQoL Work, MBSRQ Body satisfaction, EAMS, all but one of the AQoL measures, PHQ, GAD, and BPIP (higher on the first four, lower on the remaining outcomes).

**Table S1**

*Means, Standard Deviations, and Internal Consistencies of Measures Used*

| **Measures** | ***n*** | ***M (SD)*** | ***α*** | **Measures** | ***n*** | ***M (SD)*** | ***α*** | **Measures** | ***n*** | ***M (SD)*** | ***α*** |
| --- | --- | --- | --- | --- | --- | --- | --- | --- | --- | --- | --- |
| POTS upset  POTS freq. | 202  205 | 3.087 (1.757)  2.191 (1.045) | .994  .930 | AQoL SD M | 211 | 0.271 (0.153) | .918 | DASS D | 214 | 12.81 (10.07) | .900 |
| EWS | 211 | 2.164 (0.929) | .755 | AQoL SD PH | 211 | 0.581 (0.211) | .823 | DASS T | 214 | 36.24 (24.734) | .935 |
| WBIS | 213 | 5.021 (1.227) | .860 | AQoL IL | 213 | 0.778 (0.159) | .691 | PHQ | 207 | 9.6 (5.75) | .859 |
| EDE-Q G | 214 | 2.721 (1.01) | .809 | AQoL HA | 212 | 0.691 (0.154) | .582 | TOMS-2 | 212 | 2.797 (0.465) | .682 |
| EDE-Q R | 214 | 1.631 (1.355) | .768 | AQoL MH | 213 | 0.54 (0.117) | .755 | RSE | 214 | 17.3 (5.855) | .881 |
| EDE-Q EC | 214 | 1.502 (1.659) | .563 | AQoL CO | 212 | 0.659 (0.138) | .536 | MBSRQ AE | 214 | 1.84 (0.613) | .697 |
| EDE-Q SC | 214 | 3.806 (1.095) | .707 | AQoL RE | 213 | 0.646 (0.145) | .778 | MBSRQ AO | 214 | 3.52 (0.602) | .791 |
| EDE-Q WC | 214 | 3.944 (1.276) | .718 | AQoL SW | 212 | 0.698 (0.186) | .549 | MBSRQ FE | 214 | 3.01 (0.836) | .635 |
| DEBQ R | 211 | 2.676 (0.593) | .864 | AQoL PA | 211 | 0.632 (0.254) | .542 | MBSRQ FO | 214 | 2.68 (0.619) | .763 |
| DEBQ EmE | 211 | 3.228 (0.941) | .961 | AQoL SE | 213 | 0.853 (0.121) | .524 | MBSRQ HE | 214 | 2.71 (0.673) | .588 |
| DEBQ ExE | 213 | 3.49 (0.725) | .912 | IWQoL (T) | 214 | 89.935 (23.625) | .937 | MBSRQ HO | 214 | 2.94 (0.583) | .613 |
| TFEQ CR | 213 | 9.075 (2.109) | .771 | IWQoL PF | 213 | 35.352 (10.23) | .907 | MBSRQ IO | 214 | 2.94 (0.765) | .674 |
| TFEQ D | 213 | 10.38 (2.176) | .790 | IWQoL SE | 213 | 21.887 (6.656) | .920 | MBSRQ BS | 214 | 2.22 (0.495) | .715 |
| TFEQ H | 213 | 8.803 (2.784) | .849 | IWQoL SL | 209 | 11.909 (5.339) | .936 | MBSRQ OP | 214 | 3.26 (0.694) | .725 |
| CIA | 213 | 20.962 (11.503) | .946 | IWQoL PD | 214 | 12.593 (5.534) | .873 | MBSRQ WC | 214 | 4.75 (0.517) | .803 |
| QEWP | 214 | 20.014 (9.262) | .835 | IWQoL WO | 208 | 8.99 (4.117) | .834 | BPI P | 200 | 3.57 (2.088) | .909 |
| WEL | 211 | 4.368 (1.632) | .928 | GAD-7 | 208 | 5.635 (4.472) | .882 | BPI I | 192 | 4.17 (2.701) | .940 |
| EAMS | 213 | 4.302 (1.479) | .833 | DASS A | 214 | 9.355 (7.914) | .785 | - | - | - | - |
| AQoL (T) | 210 | 0.564 (0.192) | .942 | DASS S | 214 | 14.075 (9.641) | .878 | - | - | - | - |
| *Note.* All acronyms listed in correlation tables. | | | | | | | | | | | |

**Table S2**

*Results from the Hochberg Procedure, Separated by Conceptual Domain*

| **Domain** | **Test #** | **Variable** | **Observed *p*-value** | ***p*-value threshold** | **Results** |
| --- | --- | --- | --- | --- | --- |
| Disordered Eating | 1 | DEBQ-R | .229 | .050 | Not significant |
|  | 2 | DEBQ-Ext | .195 | .025 | Not significant |
|  | 3 | TFEQ-CR | .149 | .017 | Not significant |
|  | 4 | TFEQ-H | .053 | .013 | Not significant |
|  | 5 | EDEQ-R | .049 | .010 | Not significant |
|  | 6 | WEL (restraint) | .036 | .008 | Not significant |
|  | 7 | EDEQ-EC | .032 | .007 | Not significant |
|  | 8 | QEWP | .016 | .006 | Not significant |
|  | 9 | TFEQ-D | .002 | .006 | Significant |
|  | 10 | EDEQ-SC | .001 | .005 | Significant |
|  | 11 | CIA | <.001 | .005 | Significant |
|  | 12 | DEBQ-Emo | <.001 | .004 | Significant |
|  | 13 | EDEQ-WC | <.001 | .004 | Significant |
|  | 14 | EDEQ-G | <.001 | .004 | Significant |
| Psychological QoL | 1 | IWQoL(W) | .014 | .050 | Significant |
|  | 2 | IWQoL(PD) | .004 | .025 | Significant |
|  | 3 | AQoL(CO) | .003 | .017 | Significant |
|  | 4 | AQoL(HA) | .001 | .013 | Significant |
|  | 5 | AQoL(RE) | .001 | .010 | Significant |
|  | 6 | IWQoL(SE) | <.001 | .008 | Significant |
|  | 7 | IWQoL(SL) | <.001 | .007 | Significant |
|  | 8 | AQoL(MH) | <.001 | .006 | Significant |
|  | 9 | AQoL(SW) | <.001 | .006 | Significant |
| Psych Distress | 1 | DASS(A) | .003 | .050 | Significant |
|  | 2 | GAD-7 | .003 | .025 | Significant |
|  | 3 | DASS(S) | .001 | .017 | Significant |
|  | 4 | TOMS | 0.001 | .013 | Significant |
|  | 5 | RSE | <.001 | .010 | Significant |
|  | 6 | PHQ | <.001 | .008 | Significant |
|  | 7 | DASS(D) | <.001 | .007 | Significant |
| Body Image | 1 | MBSRQ IO | .836 | .050 | Not significant |
|  | 2 | MBSRQ FO | .255 | .025 | Not significant |
|  | 3 | MBSRQ FE | .019 | .017 | Not significant |
|  | 4 | MBSRQ WC | .015 | .013 | Not significant |
|  | 5 | MBSRQ HO | .011 | .010 | Not significant |
|  | 6 | MBSRQ AO | .006 | .008 | Significant |
|  | 7 | MBSRQ HE | .004 | .007 | Significant |
|  | 8 | MBSRQ OP | .003 | .006 | Significant |
|  | 9 | MBSRQ BS | .001 | .006 | Significant |
|  | 10 | MBSRQ AE | <.001 | .005 | Significant |
| Physical Health | NA |  | |  | All non-significant |

*Note.* *p-*values presented here are for the standardised effect size estimate of the indirect effect. Please see Cao and Zhang (2014) for a complete description of this procedure. From the Methods section: First, *p*-values of interest are ordered from largest to smallest. If the largest *p­*-value is > .05, the procedure sequentially reduces the parameters of significance by dividing the criterion (*α* = .05) by each additional number of comparisons, where the first comparison begins at *α* = .05, the second comparison is .05/2 (*α* = .025), the third is .05/3 (*α* = .017) and so on until the comparison reaches significance, if at all. If a comparison reaches significance in this procedure, all subsequent *p*-values are considered significant. If the largest *p*-value in the dataset is < .05, all tests are considered significant and the steps described above irrelevant.

| **Table S3**  Correlations Between BMI, Perceived Weight Stigma, and Internalised Weight Stigma and All Psychosocial and Physical Health Outcome Variables in the Current Study | | | |
| --- | --- | --- | --- |
|  | 1. BMI | 2. POTS | 3. WBIS |
| 2. POTS (Perceived Weight Stigma) | 0.14 | - |  |
| 3. WBIS (Internalised Weight Stigma) | 0.02 | 0.24*** | - |
| 3. EDE-Q Global | -0.15* | 0.25*** | 0.49*** |
| 4. EDE-Q Restraint | 0.03 | 0.12 | 0.16* |
| 5. EDE-Q Eating Concern | -0.25*** | 0.15* | 0.20** |
| 6. EDE-Q Shape Concern | -0.09 | 0.24*** | 0.55*** |
| 7. EDE-Q Weight Concern | -0.12 | 0.25*** | 0.65*** |
| 8. DEBQ Restraint | -0.03 | -0.04 | 0.12 |
| 9. DEBQ Emotional Eating | -0.10 | 0.07 | 0.42*** |
| 10. DEBQ External Eating | -0.12 | 0.19** | 0.14* |
| 11. TFEQ Cognitive Restraint | 0.02 | -0.14* | -0.10 |
| 12. TFEQ Disinhibition | -0.05 | 0.16* | 0.21** |
| 13. TFEQ Hunger | -0.17* | 0.24*** | 0.22** |
| 14. Clinical Impairment Assessment | -0.07 | 0.24*** | 0.67*** |
| 15. Questionnaire on Eating and Weight Patterns | -0.12 | 0.12 | 0.24*** |
| 16. Weight Efficacy Lifestyle Questionnaire | 0.10 | -0.11 | -0.22** |
| 17. AQoL8DUtility (Total) | -0.20** | -0.20** | -0.45*** |
| 18. Mental (Super) Dimension | -0.13 | -0.20** | -0.58*** |
| 19. Physical (Super) Dimension | -0.27*** | -0.19** | -0.02 |
| 20. Independent Living | -0.44*** | -0.24*** | -0.01 |
| 21. Happiness | -0.14* | -0.17* | -0.40*** |
| 22. Mental Health | -0.03 | -0.16* | -0.48*** |
| 23. Coping | -0.08 | -0.20** | -0.40*** |
| 24. Relationships | -0.19** | -0.28*** | -0.51*** |
| 25. Self-Worth | -0.17* | -0.27*** | -0.63*** |
| 26. Pain | -0.21** | -0.12 | -0.02 |
| 27. Senses | -0.02 | -0.14* | -0.04 |
| 28. IWQoL (lite) total | 0.35*** | 0.40*** | 0.49*** |
| 29. IWQoL physical function | 0.37*** | 0.24*** | 0.11 |
| 30. IWQoL Self-Esteem | 0.07 | 0.28*** | 0.76*** |
| 31. IWQoL Sexual Life | 0.11 | 0.15* | 0.42*** |
| 32. IWQoL Public Distress | 0.53*** | 0.53*** | 0.33*** |
| 33. IWQoL Work | 0.23** | 0.37*** | 0.33*** |
| 34. Depression Anxiety Stress Scale total | -0.01 | 0.29*** | 0.48*** |
| 35. DASS Anxiety | 0.03 | 0.37*** | 0.36*** |
| 36. DASS Stress | -0.09 | 0.17* | 0.40*** |
| 37. DASS Depression | 0.04 | 0.27*** | 0.53*** |
| 38. Patient Health Questionnaire (Depression) | -0.02 | 0.30*** | 0.49*** |
| 39. Generalised Anxiety Disorder Questionnaire | -0.04 | 0.27*** | 0.35*** |
| 40. Tolerance of Mood States (subscale 2) | -0.01 | 0.27*** | 0.52*** |
| 41. Rosenberg Self Esteem Scale | -0.05 | -0.25*** | -0.63*** |
| 42. Exercise-Avoidance Motivation Scale | 0.16* | 0.40*** | 0.60*** |
| 42. MBSRQ Appearance Evaluation | -0.18* | -0.18** | -0.50*** |
| 43. MBSRQ Appearance Orientation | -0.28*** | -0.17* | 0.21** |
| 44. MBSRQ Fitness Evaluation | -0.08 | -0.14 | -0.23*** |
| 45. MBSRQ Fitness Orientation | -0.27*** | -0.08 | -0.07 |
| 46. MBSRQ Health Evaluation | -0.22** | -0.18** | -0.32*** |
| 47. MBSRQ Health Orientation | -0.24*** | -0.26*** | -0.21** |
| 48. MBSRQ Illness Orientation | -0.04 | -0.12 | -0.04 |
| 49. MBSRQ Body Areas Satisfaction | -0.16* | -0.20** | -0.48*** |
| 50. MBSRQ Overweight Preoccupation | -0.16* | -0.03 | 0.29*** |
| 51. MBSRQ Weight Concern | 0.35*** | 0.09 | 0.26*** |
| 52. Brief Pain Inventory (Pain Severity) | 0.17* | 0.18* | -0.03 |
| 53. Brief Pain Inventory (Pain Interference) | 0.15 | 0.14* | 0.07 |
| *Note. ***p* < .001, ** *p* < .01, **p* < .05. EDE-Q = Eating Disorder Examination Questionnaire, DEBQ = Dutch Eating Behaviour Questionnaire, TFEQ = Three-Factor Eating Questionnaire, AQoL = Assessment of Quality of Life, IWQoL = Impact of Weight on Quality of Life, and MBSRQ = Multi-Dimensional Body-Self Relations Questionnaire. | | | |

| **Table S4**  Unstandardised and Standardised Regression Coefficients for the Total Effects, Each Pathway of the Mediation, and the Indirect Effect for Each Eating Behaviour Outcome Variable | | | | | | | | | | | | | | | | | | | | | |  |
| --- | --- | --- | --- | --- | --- | --- | --- | --- | --- | --- | --- | --- | --- | --- | --- | --- | --- | --- | --- | --- | --- | --- |
| **Outcome Variables** | **c (SE)** | **β c** | **c’ (SE)** | | **β c’** | | **a (SE)** | | **β a** | | **b (SE)** | | **β b** | | **a*b (SE)** | | **95% CIs** | | **β a*b** | |  |  |
| EDE-Q (Global) | 0.157 (0.041) | 0.265*** | 0.056 (0.041) | | 0.094 | | 0.252 (0.057) | | 0.363*** | | 0.400 (0.055) | | 0.471*** | | 0.101 (0.024) | | 0.058, 0.156 | | 0.171*** | | |  |
| Restraint | 0.047 (0.058) | 0.060 | -0.001 (0.065) | | -0.001 | | 0.252 (0.056) | | 0.363*** | | 0.194 (0.086) | | 0.171* | | 0.049 (0.023) | | 0.008, 0.105 | | 0.062* | | |  |
| Eating Concern | 0.111 (0.069) | 0.113 | 0.038 (0.071) | | 0.038 | | 0.252 (0.053) | | 0.363*** | | 0.289 (0.104) | | 0.204** | | 0.073 (0.029) | | 0.022, 0.142 | | 0.074* | | |  |
| Shape Concern | 0.213 (0.044) | 0.335*** | 0.098 (0.042) | | 0.153* | | 0.252 (0.056) | | 0.363*** | | 0.458 (0.068) | | 0.501*** | | 0.115 (0.028) | | 0.067, 0.181 | | 0.182*** | | |  |
| Weight Concern | 0.256 (0.051) | 0.347*** | 0.089 (0.046) | | 0.121 | | 0.252 (0.054) | | 0.363*** | | 0.660 (0.067) | | 0.622*** | | 0.167 (0.038) | | 0.097, 0.247 | | 0.226*** | | |  |
| Dutch Eating Behaviour Questionnaire | | | |  | |  | |  | |  | |  | |  | |  | |  | |  | | |
| Restraint | 0.031 (0.025) | 0.092 | 0.021 (0.027) | | 0.062 | | 0.252 (0.056) | | 0.362*** | | 0.040 (0.040) | | 0.082 | | 0.010 (0.010) | | -0.008, 0.033 | | 0.029 | | |  |
| Emotional Eating | 0.186 (0.038) | 0.338*** | 0.107 (0.040) | | 0.196** | | 0.252 (0.056) | | 0.362*** | | 0.310 (0.054) | | 0.394*** | | 0.078 (0.020) | | 0.044, 0.126 | | 0.142*** | | |  |
| External Eating | 0.102 (0.029) | 0.246*** | 0.088 (0.031) | | 0.211** | | 0.252 (0.056) | | 0.363*** | | 0.058 (0.046) | | 0.097 | | 0.014 (0.012) | | -0.006, 0.043 | | 0.035 | | |  |
| Three-Factor Eating Questionnaire | | | |  | |  | |  | |  | |  | |  | |  | |  | |  | | |
| Cognitive Restraint | -0.024 (0.157) | -0.011 | -0.115 (0.171) | | -0.051 | | 0.243 (0.053) | | 0.352*** | | 0.373 (0.236) | | 0.115 | | 0.091 (0.059) | | -0.014, 0.227 | | 0.040 | | |  |
| Disinhibition | 0.440 (0.129) | 0.232*** | 0.207 (0.143) | | 0.109 | | 0.243 (0.053) | | 0.352*** | | 0.953 (0.201) | | 0.347*** | | 0.232 (0.074) | | 0.117, 0.415 | | 0.122** | | |  |
| Hunger | 0.371 (0.157) | 0.163* | 0.249 (0.173) | | 0.110 | | 0.244 (0.050) | | 0.352*** | | 0.498 (0.250) | | 0.151* | | 0.121 (0.068) | | 0.009, 0.28 | | 0.053 | | |  |
| Clinical Impairment Assment | 2.228 (0.469) | 0.332*** | 0.697 (0.402) | | 0.104 | | 0.250 (0.055) | | 0.360*** | | 6.114 (0.581) | | 0.632*** | | 1.530 (0.339) | | 0.895, 2.230 | | 0.228*** | | |  |
| Q on Eating and Weight Ps | 0.822 (0.389) | 0.152* | 0.272 (0.4) | | 0.050 | | 0.252 (0.055) | | 0.363*** | | 2.177 (0.573) | | 0.280*** | | 0.550 (0.186) | | 0.251, 1.001 | | 0.101** | | |  |
| Weight-Efficacy Lifestyle Q | -0.081 (0.067) | -0.088 | 0.002 (0.076) | | 0.002 | | 0.249 (0.055) | | 0.358*** | | -0.334 (0.114) | | -0.253** | | -0.083 (0.036) | | -0.172, -0.027 | | -0.090* | | |  |
| *Note. c* = total effect, *c’* = direct effect, *a* = predictor-mediator relationship, *b* = mediator-outcome relationship, *a*b* = indirect effect. ****p* < .001, ***p* < .01, **p* < .05. All mediations reported are controlling for BMI. Please see Table 1 for measure acronyms in Tables 4-6. | | | | | | | | | | | | | | | | | | | | | |  |

| **Table S5**  Unstandardised and Standardised Regression Coefficients for the Total Effects, Each Pathway of the Mediation, and the Indirect Effect for Each Quality of Life Outcome Variable | | | | | | | | | | | |
| --- | --- | --- | --- | --- | --- | --- | --- | --- | --- | --- | --- |
| **Outcome Variables** | **c (SE)** | **β c** | **c’ (SE)** | **β c’** | **a (SE)** | **β a** | **b (SE)** | **β b** | **a*b (SE)** | **95% CIs** | **β a*b** |
| Assessment of QoL (Total) | -0.023 (0.008) | -0.203** | -0.006 (0.008) | -0.059 | 0.234 (0.055) | 0.341*** | -0.069 (0.012) | -0.422*** | -0.016 (0.004) | -0.026, -0.008 | -0.143*** |
| AQoL Mental health | -0.021 (0.006) | -0.242*** | -0.004 (0.006) | -0.044 | 0.249 (0.057) | 0.358*** | -0.071 (0.009) | -0.550*** | -0.017 (0.004) | -0.027, -0.009 | -0.197*** |
| Happiness | -0.014 (0.006) | -0.157* | -0.001 (0.006) | -0.019 | 0.248 (0.055) | 0.357*** | -0.049 (0.009) | -0.386*** | -0.012 (0.003) | -0.020, -0.006 | -0.138*** |
| Mental health | -0.015 (0.004) | -0.224** | -0.004 (0.005) | -0.061 | 0.252 (0.057) | 0.363*** | -0.044 (0.007) | -0.450*** | -0.011 (0.002) | -0.017, -0.006 | -0.163*** |
| Coping | -0.011 (0.005) | -0.142 | 0.000 (0.006) | -0.001 | 0.253 (0.056) | 0.363*** | -0.044 (0.009) | -0.388*** | -0.011 (0.003) | -0.019, -0.005 | -0.141*** |
| Relationships | -0.024 (0.005) | -0.295*** | -0.010 (0.005) | -0.125 | 0.252 (0.056) | 0.363*** | -0.056 (0.008) | -0.469*** | -0.014 (0.003) | -0.023, -0.007 | -0.170*** |
| Self-worth | -0.026 (0.007) | -0.240*** | -0.001 (0.007) | -0.016 | 0.253 (0.055) | 0.363*** | -0.096 (0.010) | -0.616*** | -0.024 (0.005) | -0.035, -0.014 | -0.224*** |
| AQoL Physical health | -0.009 (0.008) | -0.076 | -0.009 (0.010) | -0.075 | 0.233 (0.055) | 0.340*** | 0.000 (0.013) | -0.003 | 0.000 (0.003) | -0.007, 0.006 | -0.001 |
| Independent Living | -0.005 (0.006) | -0.061 | -0.005 (0.006) | -0.066 | 0.252 (0.056) | 0.363*** | 0.001 (0.010) | 0.013 | 0.000 (0.002) | -0.004, 0.005 | 0.005 |
| Pain | -0.010 (0.010) | -0.074 | -0.010 (0.011) | -0.069 | 0.233 (0.053) | 0.341*** | -0.003 (0.016) | -0.015 | -0.005 (0.004) | -0.009, 0.006 | -0.005 |
| Senses | 0.000 (0.005) | -0.010 | 0.000 (0.005) | 0.006 | 0.252 (0.054) | 0.363*** | -0.004 (0.008) | -0.046 | -0.001 (0.002) | -0.006, 0.002 | -0.016 |
| Impact of Weight on QoL (Total) | 4.518 (0.901) | 0.330*** | 2.255 (0.797) | 0.164** | 0.252 (0.056) | 0.363*** | 8.950 (1.222) | 0.455*** | 2.263 (0.587) | 1.223, 3.522 | 0.165*** |
| Physical Function | 0.556 (0.406) | 0.095 | 0.335 (0.471) | 0.057 | 0.252 (0.055) | 0.362*** | 0.876 (0.654) | 0.104 | 0.221 (0.178) | -0.069, 0.644 | 0.037 |
| Self-Esteem | 1.470 (0.266) | 0.377*** | 0.418 (0.195) | 0.107* | 0.252 (0.055) | 0.362*** | 4.165 (0.298) | 0.746*** | 1.051 (0.229) | 0.617, 1.525 | 0.270*** |
| Sexual Life | 0.704 (0.226) | 0.226** | 0.209 (0.216) | 0.067 | 0.253 (0.058) | 0.363*** | 1.951 (0.291) | 0.439*** | 0.495 (0.136) | 0.265, 0.811 | 0.159*** |
| Public Distress | 1.170 (0.179) | 0.371*** | 0.903 (0.172) | 0.286*** | 0.252 (0.056) | 0.363*** | 1.056 (0.260) | 0.233*** | 0.267 (0.089) | 0.124, 0.483 | 0.084** |
| Work | 0.667 (0.163) | 0.287*** | 0.443 (0.163) | 0.190** | 0.248 (0.056) | 0.357*** | 0.905 (0.250) | 0.270*** | 0.224 (0.084) | 0.094, 0.432 | 0.096** |
| *Note*. *c* = total effect, *c’* = direct effect, *a* = predictor-mediator relationship, *b* = mediator-outcome relationship, *a*b* = indirect effect. ****p* < .001, ***p* < .01, **p* < .05. All mediations reported are controlling for BMI. | | | | | | | | | | | |

| **Table 6**  Unstandardised and Standardised Regression Coefficients for the Total Effects, Each Pathway of the Mediation, and the Indirect Effect for Each Anxiety, Body Image, and other Psychosocial and Physical Outcome Variables | | | | | | | | | | | | | | | | | | | | | | | | | | | | | |  |
| --- | --- | --- | --- | --- | --- | --- | --- | --- | --- | --- | --- | --- | --- | --- | --- | --- | --- | --- | --- | --- | --- | --- | --- | --- | --- | --- | --- | --- | --- | --- |
| **Outcome Variables** | | **c (SE)** | | | **β c** | **c’ (SE)** | | | | | **β c’** | **a (SE)** | | | **β a** | | | **b (SE)** | **β b** | | | **a*b (SE)** | | | **95% CIs** | | | **β a*b** | | |
| Depression Anxiety Stress Scale | |  | |  | | | |  | |  | | | |  | | |  |  | | |  | | |  | | |  | |  |  |
| Anxiety | | 1.267 (0.326) | | | 0.276*** | | 0.706 (0.315) | | | | 0.154* | | 0.252 (0.057) | | | 0.363*** | | 2.220 (0.456) | | 0.337*** | | | 0.561 (0.172) | | | 0.256, 0.950 | | 0.122*** | |  |
| Stress | | 1.267 (0.326) | | | 0.276*** | | 0.706 (0.315) | | | | 0.154* | | 0.252 (0.057) | | | 0.363*** | | 2.22 (0.456) | | 0.337*** | | | 0.561 (0.172) | | | 0.256, 0.950 | | 0.122*** | |  |
| Depression | | 1.265 (0.426) | | | 0.214** | | 0.183 (0.467) | | | | 0.031 | | 0.252 (0.056) | | | 0.363*** | | 4.280 (0.638) | | 0.505*** | | | 1.082 (0.266) | | | 0.609, 1.662 | | 0.183*** | |  |
| Patient Health Questionnaire | | 0.668 (0.242) | | | 0.202** | | 0.111 (0.253) | | | | 0.033 | | 0.245 (0.056) | | | 0.353*** | | 2.265 (0.370) | | 0.476*** | | | 0.556 (0.162) | | | 0.275, 0.920 | | 0.168*** | |  |
| Generalised Anxiety Disorder Assment | | 0.447 (0.187) | | | 0.175* | | 0.121 (0.205) | | | | 0.047 | | 0.246 (0.054) | | | 0.354*** | | 1.326 (0.287) | | 0.361*** | | | 0.326 (0.101) | | | 0.142, 0.565 | | 0.128*** | |  |
| Tolerance of Mood Scale | | 0.105 (0.018) | | | 0.390*** | | 0.062 (0.018) | | | | 0.231*** | | 0.252 (0.055) | | | 0.363*** | | 0.169 (0.027) | | 0.436*** | | | 0.042 (0.011) | | | 0.023, 0.068 | | 0.158*** | |  |
| Rosenberg Self-Esteem Scale | | -0.943 (0.244) | | | -0.273*** | | -0.186 (0.230) | | | | -0.054 | | 0.252 (0.056) | | | 0.363*** | | -2.995 (0.304) | | -0.605*** | | | -0.757 (0.174) | | | -1.145, -0.449 | | -0.219*** | |  |
| Exercise-Avoidance Motivation Scale | | 0.354 (0.056) | | | 0.417*** | | 0.199 (0.056) | | | | 0.234*** | | 0.252 (0.055) | | | 0.363*** | | 0.613 (0.092) | | 0.503*** | | | 0.155 (0.038) | | | 0.089, 0.239 | | 0.183*** | |  |
| Multi-Dimensional Body-Self Relations Questionnaire | | | | | |  | | |  | | |  | | |  | | |  |  | | |  | | |  | | |  | | |
| Appearance Evaluation | -0.078 (0.025) | | -0.223** | | | -0.016 (0.023) | | | -0.047 | | | 0.252 (0.055) | | | 0.363*** | | | -0.246 (0.032) | -0.486*** | | | -0.062 (0.016) | | | -0.100, -0.034 | | | -0.176*** | | |
| Appearance Orientation | 0.036 (0.023) | | 0.110 | | | 0.005 (0.024) | | | 0.017 | | | 0.252 (0.055) | | | 0.363*** | | | 0.123 (0.033) | 0.256*** | | | 0.031 (0.011) | | | 0.013, 0.058 | | | 0.092** | | |
| Fitness Evaluation | -0.041 (0.036) | | -0.083 | | | 0.001 (0.040) | | | 0.003 | | | 0.252 (0.055) | | | 0.363*** | | | -0.170 (0.055) | -0.239** | | | -0.042 (0.017) | | | -0.084, -0.015 | | | -0.086* | | |
| Fitness Orientation | -0.002 (0.025) | | -0.006 | | | 0.007 (0.027) | | | 0.019 | | | 0.252 (0.059) | | | 0.363*** | | | -0.037 (0.037) | -0.072 | | | -0.009 (0.009) | | | -0.033, 0.007 | | | -0.026 | | |
| Health Evaluation | -0.049 (0.027) | | -0.128 | | | -0.004 (0.031) | | | -0.011 | | | 0.252 (0.056) | | | 0.363*** | | | -0.179 (0.038) | -0.322*** | | | -0.045 (0.014) | | | -0.078, -0.021 | | | -0.117** | | |
| Health Orientation | -0.079 (0.023) | | -0.237*** | | | -0.055 (0.022) | | | -0.165* | | | 0.252 (0.055) | | | 0.363*** | | | -0.095 (0.032) | -0.198** | | | -0.024 (0.009) | | | -0.048, -0.009 | | | -0.072** | | |
| Illness Orientation | -0.024 (0.032) | | -0.057 | | | -0.024 (0.033) | | | -0.057 | | | 0.252 (0.056) | | | 0.363*** | | | 0.000 (0.049) | 0.000 | | | 0.000 (0.012) | | | -0.025, 0.025 | | | 0.000 | | |
| Body Areas Satisfaction | -0.058 (0.020) | | -0.204** | | | -0.011 (0.019) | | | -0.040 | | | 0.252 (0.055) | | | 0.363*** | | | -0.185 (0.028) | -0.451*** | | | -0.046 (0.012) | | | -0.074, -0.026 | | | -0.163*** | | |
| Overweight Preoccupation | 0.012 (0.028) | | 0.031 | | | -0.033 (0.026) | | | -0.085 | | | 0.252 (0.055) | | | 0.363*** | | | 0.183 (0.039) | 0.322*** | | | 0.046 (0.013) | | | 0.023, 0.078 | | | 0.116*** | | |
| Self-Classified Weight | 0.030 (0.016) | | 0.125 | | | 0.006 (0.019) | | | 0.027 | | | 0.252 (0.056) | | | 0.363*** | | | 0.094 (0.024) | 0.270*** | | | 0.023 (0.008) | | | 0.009, 0.043 | | | 0.098** | | |
| Brief Pain Inventory |  | |  | | |  | | |  | | |  | | |  | | |  |  | | |  | | |  | | |  | | |
| Pain Interference | 0.146 (0.119) | | 0.094 | | | 0.115 (0.129) | | | 0.074 | | | 0.253 (0.058) | | | 0.367*** | | | 0.122 (0.190) | 0.054 | | | 0.031 (0.049) | | | -0.062, 0.136 | | | 0.020 | | |
| Pain Severity | 0.009 (0.092) | | 0.008 | | | 0.022 (0.098) | | | 0.018 | | | 0.251 (0.057) | | | 0.363*** | | | -0.050 (0.148) | -0.028 | | | -0.012 (0.038) | | | -0.093, 0.064 | | | -0.010 | | |
| *Note. c* = total effect, *c’* = direct effect, *a* = predictor-mediator relationship, *b* = mediator-outcome relationship, *a*b* = indirect effect. ****p* < .001, ***p* < .01, **p* < .05. All mediations reported are controlling for BMI. MBSRQ scores – low scores indicate: AE = negative body image, dissatisfied with appearance, AO = apathetic about appearance, looks viewed as not particularly important, FE = considers self unfit, does not value physical fitness nor regularly exercises, FO = does not value physical fitness nor regularly exercises, HE = feels unhealthy and ill, HO = apathetic about health, IO = not very alert to personal symptoms of physical illness, BAS = unhappy with size/appearance, OP = minimal feelings of fat anxiety, dieting/restraint, and weight vigilance, WC = low self-perceived weight. | | | | | | | | | | | | | | | | | | | | | | | | | | | | | |  |

**References**

1. Thompson JK, Cattarin J, Fowler B, Fisher E. The perception of teasing scale (POTS): A revision and extension of the physical appearance related teasing scale (PARTS). Journal of personality assessment. 1995;65(1):146-57.

2. Durso LE, Latner JD. Understanding self‐directed stigma: development of the weight bias internalization scale. Obesity. 2008;16(S2):S80-S6.

3. Pearl RL, Puhl RM. Measuring internalized weight attitudes across body weight categories: validation of the modified weight bias internalization scale. Body image. 2014;11(1):89-92.

4. Bohn K, Fairburn CG. The clinical impairment assessment questionnaire (CIA). Cognitive behavioral therapy for eating disorders. 2008:315-7.

5. Van Strien T, Frijters JE, Bergers GP, Defares PB. The Dutch Eating Behavior Questionnaire (DEBQ) for assessment of restrained, emotional, and external eating behavior. International journal of eating disorders. 1986;5(2):295-315.

6. Fairburn CG, Beglin SJ. Assessment of eating disorders: Interview or self‐report questionnaire? International journal of eating disorders. 1994;16(4):363-70.

7. Fairburn CG. Cognitive behavior therapy and eating disorders: Guilford Press; 2008.

8. Vartanian LR, Shaprow JG. Effects of weight stigma on exercise motivation and behavior: a preliminary investigation among college-aged females. Journal of health psychology. 2008;13(1):131-8.

9. Spitzer R, Yanovski S, Marcus M. The questionnaire on eating and weight patterns-revised (QEWP-R). New York: New York State Psychiatric Institute. 1993.

10. Stunkard AJ, Messick S. The three-factor eating questionnaire to measure dietary restraint, disinhibition and hunger. Journal of psychosomatic research. 1985;29(1):71-83.

11. Clark MM, Abrams DB, Niaura RS, Eaton CA, Rossi JS. Self-efficacy in weight management. Journal of consulting and clinical psychology. 1991;59(5):739.

12. Hawthorne G, Richardson J, Osborne R. The Assessment of Quality of Life (AQoL) instrument: a psychometric measure of health-related quality of life. Quality of life research. 1999;8(3):209-24.

13. Kolotkin RL, Crosby RD. Psychometric evaluation of the impact of weight on quality of life-lite questionnaire (IWQOL-lite) in a community sample. Quality of Life Research. 2002;11(2):157-71.

14. Lovibond PF, Lovibond SH. The structure of negative emotional states: Comparison of the Depression Anxiety Stress Scales (DASS) with the Beck Depression and Anxiety Inventories. Behaviour research and therapy. 1995;33(3):335-43.

15. Spitzer R, Kroenke K, Williams JB, Löwe B. A brief measure for assessing generalized anxiety disorder: the GAD-7. Archives of internal medicine. 2006;166(10):1092-7.

16. Kroenke K, Spitzer RL, Williams JB. The PHQ‐9: validity of a brief depression severity measure. Journal of general internal medicine. 2001;16(9):606-13.

17. Cash TF. Multidimensional body-self relations questionnaire: MBSRQ user’s manual. Norfolk, VA: Old Dominion University. 2000.

18. Rosenberg M. Rosenberg self-esteem scale (RSE). Acceptance and commitment therapy Measures package. 1965;61(52):18.

19. Allen KL, McLean NJ, Byrne SM. Evaluation of a new measure of mood intolerance, the Tolerance of Mood States Scale (TOMS): Psychometric properties and associations with eating disorder symptoms. Eating behaviors. 2012;13(4):326-34.

20. Cleeland CS, Ryan K. The brief pain inventory. Pain Research Group. 1991:143-7.

1. Total scores are usually calculated by including all responses except those who selected “never”. However, to account for those responding never, we coded those who responded never to all six items to be assigned a 0 so these participants would be included in the computation of total scores (and not left out as they are normally). Otherwise, scores were computed as normal. [↑](#footnote-ref-1)
